# Evaluation on the effectiveness of 2-deoxyglucose-6-phosphate phosphatase (*DOG^R^1*) gene as a selectable marker for oil palm (*Elaeis guineensis* Jacq.) embryogenic calli transformation mediated by *Agrobacterium tumefaciens*

**DOI:** 10.3389/fpls.2015.00727

**Published:** 2015-09-23

**Authors:** Abang Masli Dayang Izawati, Mat Yunus Abdul Masani, Ismail Ismanizan, Ghulam Kadir Ahmad Parveez

**Affiliations:** ^1^Advanced Biotechnology and Breeding Centre, Malaysian Palm Oil BoardSelangor, Malaysia; ^2^School of Bioscience and Biotechnology, Faculty of Science and Technology, Universiti Kebangsaan MalaysiaSelangor, Malaysia; ^3^Institute of System Biology (INBIOSIS), University Kebangsaan MalaysiaSelangor, Malaysia

**Keywords:** *DOG^R^1* gene, 2-deoxyglucose, oil palm embryogenic calli, *Agrobacterium*-mediated transformation

## Abstract

*DOG^R^1*, which encodes 2-deoxyglucose-6-phosphate phosphatase, has been used as a selectable marker gene to produce transgenic plants. In this study, a transformation vector, pBIDOG, which contains the *DOG^R^1* gene, was transformed into oil palm embryogenic calli (EC) mediated by *Agrobacterium tumefaciens* strain LBA4404. Transformed EC were exposed to 400 mg l^-1^ 2-deoxyglucose (2-DOG) as the selection agent. 2-DOG resistant tissues were regenerated into whole plantlets on various regeneration media containing the same concentration of 2-DOG. The plantlets were later transferred into soil and grown in a biosafety screenhouse. PCR and subsequently Southern blot analyses were carried out to confirm the integration of the transgene in the plantlets. A transformation efficiency of about 1.0% was obtained using *DOG^R^1* gene into the genome of oil palm. This result demonstrates the potential of using combination of *DOG^R^1* gene and 2-DOG for regenerating transgenic oil palm.

## Introduction

Oil palm (*Elaeis guineensis* Jacq.), a monocot tree, is the most important economic crop for Malaysia. Palm oil produced from this tree contributes for around 30% of total world’s vegetable oil production. Being a major crop for the country, the oil palm industry has to remain competitive by increasing yield per unit area as well as producing novel high-value products using approaches such as conventional breeding and genetic engineering (Murphy, 2014; Parveez et al., 2015a). The main target of oil palm genetic engineering is increasing the oleic acid content at the expense of palmitic acid (Ravigadevi et al., 2009). Other targets are increasing specialty fatty acids such as ricinoleic, palmitoleic, and stearic, increasing lycopene content as well as synthesizing biodegradable plastics. In order to achieve the above target, construction of transformation vectors for various products have (Yunus and Kadir, 2008; Yunus et al., 2008; Masani et al., 2009) and are being carried out before they could be used to transform oil palm. Being a perennial tree and having a long regeneration time of around 7–10 years (Rajanaidu and Jalani, 1995) the use of conventional breeding, with the need of crossing and back-crossing, will require nearly half a decade to introduce a new trait into this species. Therefore, in order to genetically engineer oil palm for modifying fatty acid composition or producing novel products, development of a reliable transformation system is essential.

Transformation of oil palm is very difficult due to two major challenges: (i) the tissue culture process, and (ii) the selection and regeneration of transgenic plants. Clonal propagation of oil palm is only feasible through callogenesis and embryogenesis which exhibit slow growth and low response under *in vitro* condition (Abdullah and Harun, 1989; Hashim et al., 2011). It was reported that the rate of callogenesis of oil palm main explants, i.e., young immature leaves, remained low, at about 19% (Corley and Tinker, 2003). Furthermore, the average rate of embryogenesis from the callus ranges from 3 to 6% where 50% of them later failed to regenerate (Wooi, 1995; Hashim et al., 2011). The variation on embryogenesis rate is random and genotype-dependent. Almost all cultured explants could produce some amount of callus, but only about 60% of the explants are capable of producing embryos. Depending on the genotype, first callus formation from oil palm explants takes as early as 3 months and can be as long as 12 months. Regeneration of oil palm plantlets, around 15 cm height, from callus stage requires additional 18–24 months (Hashim et al., 2011). For regenerating transgenic oil palm, additional 6–8 months are needed for selecting transformed cells from the majority of untransformed cells of the embryogenic calli (EC) on selection medium.

The second challenge is selection and regeneration of transgenic plants. Even though regeneration of transgenic oil palm from EC was first reported more than 10 years ago, the transformation efficiencies were rather low, between 0.7 and 1.5% only (Parveez et al., 2000; Masli et al., 2009). These reports used EC as target tissue and the efficiency was calculated based on percentage of transient over stable transformation. Therefore, more efforts should be channeled toward improving transformation efficiency. One of the approaches to increase transformation efficiency is to evaluate selection agents used to select transformed cells from the majority of untransformed cells. Transformation of oil palm has initially been carried out using herbicide Basta, as selection agents (Parveez et al., 2000). From minimal inhibition studies, it was found that Basta and hygromycin were the most efficient selection agents for oil palm transformation (Parveez et al., 1996). Kanamycin was shown to be the poorest selectable marker for oil palm as it could only kill 75% of the EC or 15% of the immature embryos at 2000 mg l^-1^ (Parveez et al., 2007). Basta has been shown to be suitable for transformation of other monocots such as wheat (Melchiorre et al., 2002), maize (Chen et al., 2008), rice (Zhao et al., 2011), and turfgrass (Song et al., 2013). Hygromycin was also reported to be used as a selection agent in other monocots such as zoysiagrass (Ge et al., 2006), finger millet (Ignacimuthu and Ceasar, 2012), and rice (Htwe et al., 2014). As common selectable markers do not improve oil palm transformation efficiency, evaluation of other alternative selectable markers are required. There are more than 50 different selectable markers that are available and ready to be evaluated for plant species (Miki and McHugh, 2004). Recently, Rosellini (2012) has classified the selectable marker genes into two selection types, (i) positive and (ii) negative, where the positive type will confer selective advantage to transgenic cells while the negative will have disadvantage to transgenic cells. Positive selection was further divided into seven types: (1) confer resistance or tolerance to phytotoxin, biotic, or abiotic stress; (2) removal of phytotoxin from the sensitive cells compartment; (3) overexpression of a sensitive or insensitive target molecule; (4) resistance to pathogen; (5) tolerance to heat; (6) carbohydrate metabolism, and (7) metabolism of growth regulators. Some examples of non-common positive selection agents are mannose (Joersbo et al., 1998), xylose (Haldrup et al., 1998), and deoxyglucose or 2-DOG (Kunze et al., 2001), which have been successfully demonstrated in a number of plants.

Besides using the common selection agent, like Basta, other non-common selection agents were also evaluated in oil palm with the aim of increasing transformation efficiency. The first non-common selection system applied to oil palm is the green fluorescence protein (GFP) which also acts as a visual selection agent (Majid and Parveez, 2007; Parveez and Majid, 2008). Good transient expression of GFP gene was detected in transformed oil palm tissues, however, so far the transformed cells failed to regenerate into any transgenic oil palm expressing GFP in the whole plant. It was expected that the use of GFP could increase transformation efficiency due to its easy visual based selection. Later, mannose based selection system was evaluated for increasing the transformation efficiency of oil palm (Bahariah et al., 2013). Mannose cannot be usually metabolized by non-transformed cells and is converted into mannose-6-phosphate by endogenous hexokinase (Joersbo and Okkels, 1996). Therefore, when mannose is added to the culture medium, it can minimize plant growth due to mannose-6-phosphate accumulation. The mannose based selection is dependent on an *E. coli pmi* gene which encodes phosphomannose isomerase (PMI) that functions to convert mannose-6-phosphate into fructose-6-phosphate (Miles and Guest, 1984). Fructose-6-phosphate could then be immediately incorporated into the plant metabolic pathway, and thus allows the mannose to be used as the sole source of carbohydrate by the transformed cells (Reed et al., 2001). In this selection system, oil palm cultures were transformed with *pmi* gene that allows the oil palm EC to utilize mannose as a carbon source and subsequently resulted in regeneration of transgenic oil palm plantlets. The result indicates that the mannose-based selection system can be used as an alternative to antibiotic and herbicide selection systems for oil palm transformation. However, due to its nature of selection, causing the untransformed cells to starve instead of killing them, the identification of single events is difficult thus complicating calculation of transformation efficiency. This also resulted in regeneration of many escape plants due to ineffective selection system.

After failing to improve the efficiency of oil palm transformation using various selection agents, such as Basta, GFP and mannose, efforts were continued to evaluate effectiveness of another selection agent, 2-deoxyglucose (2-DOG). In plant cells, 2-DOG (a glucose analog), is phosphorylated by hexokinase to form 2-DOG-6-phosphate, which competes with glucose-6-phosphate causing cell death through the inhibition of glycolysis, protein synthesis, cell wall polysaccharide synthesis and also protein glycosylation (Zemek etal., 1976). *DOG^R^1* gene, which has been isolated from *Saccharomyces cerevisiae* strain S288C, encodes 2-deoxyglucose-6-phosphate. The enzyme has the ability to convert toxic 2-DOG-6-phosphate to non-toxic products through dephosphorylation (Kunze et al., 2001). Therefore, transformed plant cells which carry the *DOG^R^1* gene can be selected on medium containing 2-DOG. Even though 2-DOG can cause a number of unknown biochemical changes in plant cells, transgenic plants regenerated were phenotypically normal and fertile. The use of 2-DOG as selection agent has resulted in successful regeneration of transgenic plants for pea (Sonnewald and Ebneth, 1998), tomato, tobacco, potato (Kunze et al., 2001) and crowtoe (*Lotus corniculatus*; Guo, 2007).

In this paper we describe the efforts to evaluate the effectiveness of 2-DOG as an alternative selection agent for oil palm transformation. A transformation vector, pBIDOG containing the *DOG^R^1* gene was constructed and used to transform EC of oil palm using *Agrobacterium*-mediated transformation. Transformed oil palm cells were selected on medium containing optimal concentration of 2-DOG which has been determined earlier (Masli et al., 2012).

## Materials and Methods

### Construction of pBIDOG Plasmid

**FIGURE 1:**
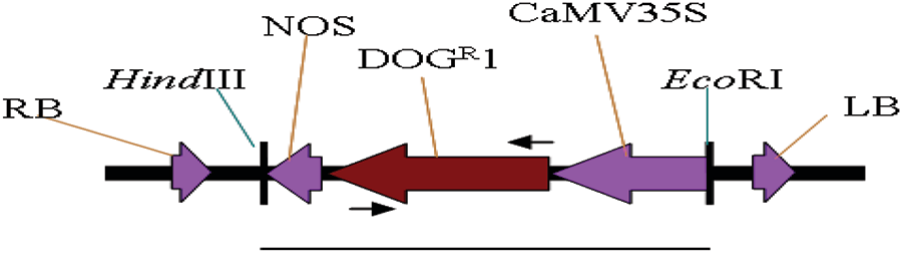
**Schematic diagram of *DOG^R^1* expression cassette (line indicates the size of 1.5 kb) used as probe for Southern blot analysis**. RB, right border; LB, left border; NOS, nopaline synthase gene terminator; *DOG^R^1*, gene codes for 2-deoxyglucose-6-phosphate phosphatase; CaMV35S, cauliflower mosaic virus 35S promoter. Arrows indicates the position of primers used for PCR analysis to amplify 741 bp.

The construction of pBIDOG plasmid was performed by replacing the DNA fragment of *CaMV35S-GUS-Nos* of pBI35SGUS with the DNA fragment of *CaMV35S-DOG^R^1-Nos* of pBINARDOG (SunGene, Germany). pBI121 plasmid was digested with *Pme*I and *Cla*I to remove the DNA fragment of *NosPro-nptII-Nos*, rendered blunt and religated to generate plasmid pBI35SGUS. The *CaMV35S-DOG^R^1-Nos* fragment was released from pBINARDOG by *Hind*III and *Eco*RI digestion, and cloned into pBI35SGUS releasing the DNA fragment of *CaMV35S-GUS-Nos* at similar sites to generate pBIDOG (**Figure 1**). pBIDOG was transformed into *E. coli* and later mobilized into *Agrobacterium tumefaciens* strain LBA4404 *via* electroporation and used as a vector for oil palm transformation.

### Plant Material and Medium

Suspension EC, initiated from oil palm immature leaves (*E. guineensis* Jacq. var Tenera) were cultured onto EC agar medium [MS salts (Murashige and Skoog, 1962); Y3 vitamins; 0.0375 g l^-1^ NaFeEDTA; 0.1 g l^-1^ myo-Inositol; 0.1 g l^-1^ L-glutamine; 0.1 g l^-1^ L-asparagine; 0.1 g l^-1^ L-arginine; 3% sucrose; 5 μM α-Naphtaleneacetic acid (NAA); 0.8% plant agar; pH 5.7] overnight. The calli were pre-treated with plasmolysis medium (PM) [MS salts; Y3 vitamins; 0.0375 g l^-1^ NaFeEDTA; 0.1 g l^-1^ myo-Inositol; 0.1 g l^-1^ L-glutamine; 0.1 g l^-1^ L-asparagine; 0.1 g l^-1^ L-arginine; 200 μM acetosyringone; 6% sucrose; 0.8% agar; pH 5.7] for 1 h prior to agroinfection.

### Agroinfection of Oil Palm Embryogenic Calli

Embryogenic calli were subjected to physical injury via bombardment prior to agroinfection. EC were bombarded with 1.0 μm gold particle without DNA using Biolistic PDS/He 1000 device (BioRad Laboratories, Hercules, CA, USA) according to Parveez et al. (1997, 1998). Fifty micro liter glycerol stock of the *A. tumefaciens* strain LBA4404 harboring pBIDOG was inoculated in 20 ml LB medium containing 50 μg ml^-1^ kanamycin and 50 μg ml^-1^ rifampicin and incubated for 2 days in the dark at 28°C. Two milliliter of the bacteria was transferred into fresh 18 ml LB medium and the culture was further incubated until the optical density (OD_600_) was between 0.2 and 0.4 nm prior to transformation. The bombarded calli were infected with the bacterium culture described above and slowly shaken on rotary shaker for 2 h. Then, the calli were blotted dry on sterile filter paper before being transferred onto PM agar medium for 24 h. Calli were subsequently transferred onto co-cultivation medium (CM) [EC agar medium; 200 μM acetosyringone; pH 5.7] for 2 days at 27°C in the dark. After 2 days, the calli were cultured on EC agar medium supplemented with 400 mg l^-1^ cefotaxime for a week. Next, the calli were transferred onto the same medium with 200 mg l^-1^ cefotaxime for 3 weeks at 28°C with 16/8 h (light/dark). Subculturing of calli was performed using the same medium supplemented with 400 mg l^-1^ 2-DOG [2-Deoxy-*D*-glucose Grade II (D8375), Sigma–Aldrich, St. Louis, USA] for 4 weeks until polyembryoid with shoot buds were formed. The shoots were then transferred into test tubes containing rooting induction medium (RIM) [MS salts; Y3 vitamins; 0.0375 g l^-1^ NaFeEDTA; 0.1 g l^-1^ myo-Inositol; 0.3 g l^-1^ L-glutamine; 9 μM NAA; 3% sucrose; 0.15% activated charcoal; 0.8% agar; pH 5.7] for development of plantlets.

### Genomic DNA Extraction, Polymerase Chain Reaction (PCR), and Southern Blot Analysis of Transgenic Plants

Genomic DNA was isolated from putative transgenic, whitish embryoids and shoots, using the modified method of Ellis (1993) and modified CTAB method (Doyle and Doyle, 1990), respectively. PCR analysis was performed to verify the presence of *DOG^R^1* gene in the genome of putative transgenic whitish embryoids. DNA was amplified using the *DOG^R^1* gene primers; DOG^R^1: 5′-ATGGATCCCCATGGCAGAATTTTCAGCTGATCTATG-3′; DOG^R^2: 5′- ATGTCGACTACTCAGGCCCTTGTCAAAGGGTTG-3′ (**Figure 1**). The PCR conditions used were as follows: the reaction mixtures were initially heated at 95°C for 5 min. Then the amplification was performed in 10 cycles at 95°C for 1 min, 70°C for 1 min and 72°C for 1 min. The annealing temperature was reduced by 1.7°C in each cycle. The reaction was continued for 25 cycles of 95°C for 1 min, 53°C for 1 min, and 72°C for 1 min. The reaction mixtures were subjected to a final extension of 72°C for 5 min. PCR products were separated by electrophoresis in 1% (w/v) agarose gels at 100 V and detected by ethidium bromide staining.

For Southern analysis, 30 μg genomic DNA of putative transgenic shoots were digested with *Eco*RI and *Hind*III and separated on 1% agarose gel in 1x TBE buffer at 30 V overnight. Copy number reconstruction contained similar amount genomic DNA of untransformed shoots (control plant) digested with *Eco*RI and *Hind*III and spiked with 0, 0.1, 0.5, and 1 copy number equivalents of the 1.5 kb T-DNA region of pBIDOG plasmid. The calculation of copy number was based on the assumption that oil palm genome size is 3.83 pg/2C (Madon et al., 2008). The separated DNA were transferred onto nylon membrane (Amersham Hybond-N^+^) by capillary blotting (Sambrook et al., 1989). The nylon membrane was pre-hybridized at 50°C for 3 h in hybridization buffer [0.5 M Na_2_HPO_4_; 0.5 M NaH_2_PO_4_; 0.5 M EDTA, pH 8.0, 7% (w/v) SDS]. Subsequently, the membrane was incubated in new hybridization buffer containing α-dATP^32^ labeled probe for overnight at 50°C. The radiolabelled probe, the *DOG^R^1* gene fragment, was prepared using pBIDOG digested with *Eco*RI and *Hind*III (**Figure 1**). The membrane was washed with 2x SSC with 0.1% (w/v) SDS at 50°C for 10 min followed by 1x SSC with 0.1% SDS at 50°C for 10 min. The membrane was exposed to an X-ray film (Kodak Photo Film) at –70°C for 7 days. The film was visualized using the standard developer and fixer solutions (Kodak).

## Results and Discussion

### Transformation and Regeneration of Transgenic Oil Palm

**FIGURE 2:**
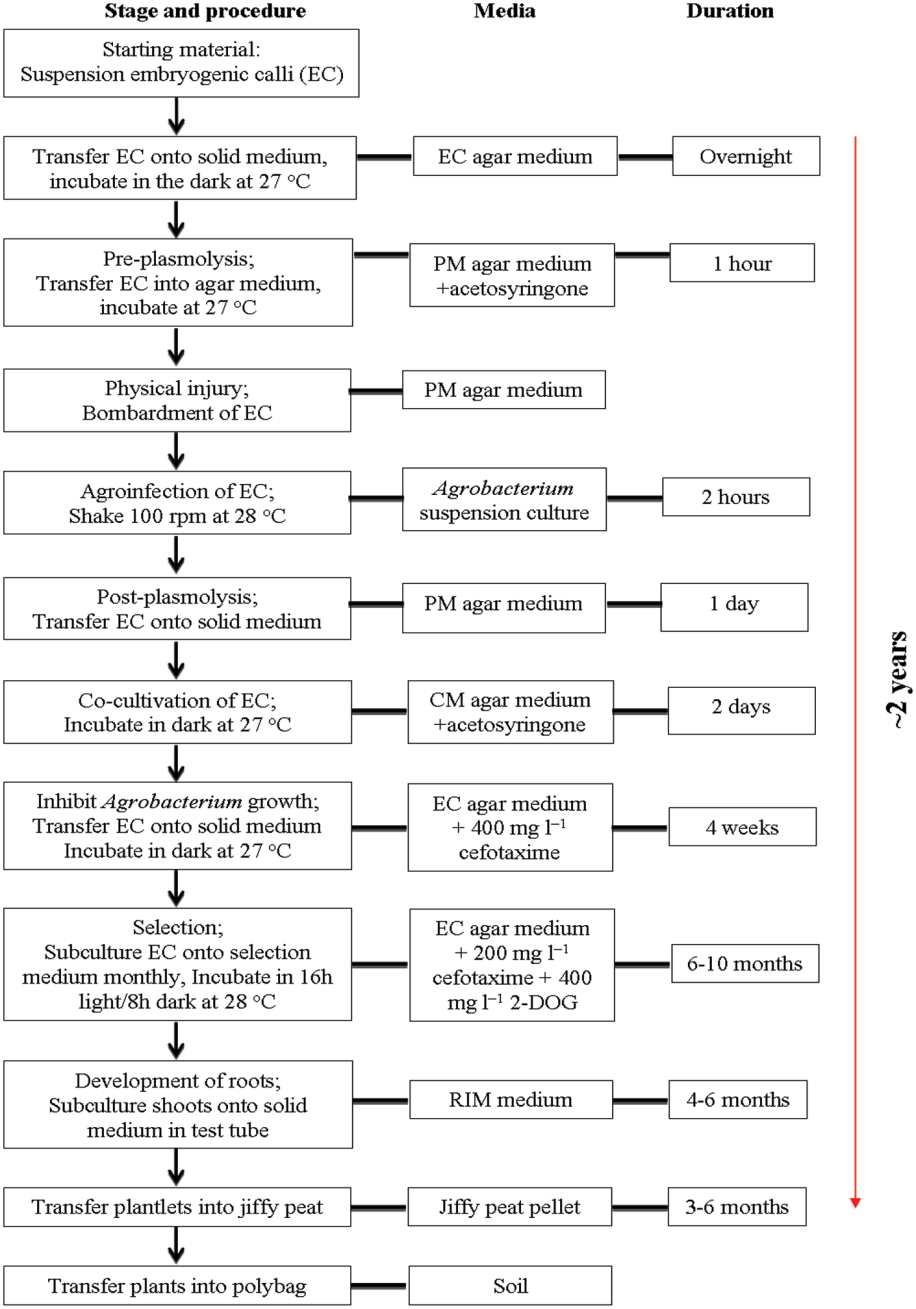
**Protocol for *Agrobacterium*-mediated transformation and regeneration of transgenic oil palm selected on 2-DOG medium**.

The simplified schematic protocol for *Agrobacterium*-mediated transformation and regeneration of transgenic oil palm selected on 2-deoxyglucose medium is summarized in **Figure 2**. In this study, suspension EC, were chosen as the starting material for oil palm transformation as they are easily regenerated into plantlets (Tarmizi et al., 2008). Embryogenic cultures were also reported to be a good target tissue as they provided a source of dividing cells that were recognized as the most competent cells for genetic transformation of pine species, cassava, sweet potato, rice, and garlic (Trontin et al., 2007; Bull et al., 2009; Zang et al., 2009; Rahman et al., 2010; Alvarez and Ordás, 2013; Lagunes-Fortiz et al., 2013). The use of highly regenerative tissue as target for transformation often results in the regeneration of a large number of independently transformed lines.

**FIGURE 3:**
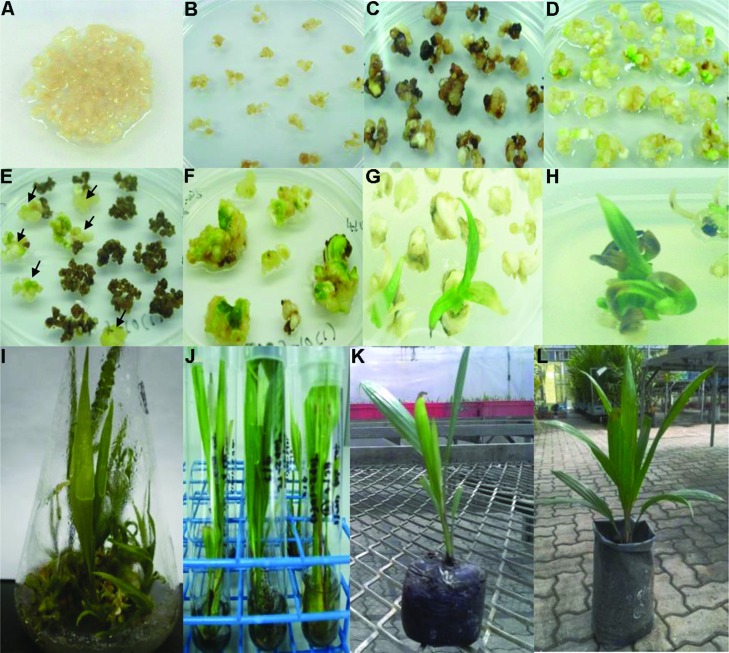
**Regeneration of transformed oil palm. (A)** Suspension embryogenic calli (EC) cultured on EC medium. **(B)** EC after infection with *Agrobacterium* suspension cultured on medium without selection. **(C)** EC in selection stage using 2-DOG medium. **(D)** Untransformed (control) EC proliferated into whitish and green embryoids when cultured on medium without selection. **(E)** Resistant EC (arrows) cultured on selection medium. **(F)** Resistant EC were separated from dead EC for proliferation into polyembryoid. **(G)** Whitish polyembryoid and shoot buds. **(H)** Shoots. **(I)** Transgenic shoots in flask for proliferation. **(J)** Transgenic shoots in rooting stage. **(K)** Plantlet with strong shoots and roots were transferred into jiffy peat pellet. **(L)** Transgenic oil palm plant in polybag.

The EC were pre-treated on a plasmolysis (PM) medium containing acetosyringone prior to bombardment (**Figure 3A**). Pre-treatment of the EC onto PM medium which contained acetosyringone helped to induce the *vir* gene transfer (Hiei et al., 1994). The function of the *vir* genes on the Ti plasmid is to facilitate excision of the T-DNA region of the plasmid and promote its transfer and integration into the plant genome. Lignin, flavanoid precursors and acetosyringone are compounds known to induce *vir* gene expression (Stachel et al., 1985). Pre-treatment by a PM containing acetosyringone has also been reported to improve transformation of other plants such as rice, orchid, garlic, *Panax ginseng*, gherkin (*Cucumis anguria*), and *Artemisia annua* and onion (Uzè et al., 1997; Choi et al., 2001; Men et al., 2003a; Kenel et al., 2010; Thiruvengadam et al., 2013; Tian et al., 2013; Xu et al., 2014). Prior to *Agrobacterium* infection, oil palm EC were bombarded with gold particles to initiate physical injury. Biolistic approach to create wounding was also reported to promote the transformation process and increase transformation efficiency in other crops such as carnation, tobacco, and rapeseed (Zuker et al., 1999; Kumar et al., 2006; Abdollahi et al., 2007). Therefore, based on the above reports, both pre-treatment and wounding were carried out with the expectation to help increase the oil palm transformation efficiency.

After infection with *Agrobacterium*, the EC were cultured onto EC medium without selection (2-DOG) for 1 month to allow for better proliferation and reduce transformation process stress (**Figure 3B**). Cefotaxime at 400 mg l^-1^ was added into the mixture to kill the excess *Agrobacterium*. Delaying exposure to a selection agent is often carried out in order to provide time for the single transgenic cell to divide a few times and increase its ability to express the transgene in the presence of selection pressure as demonstrated in peanut and maritime pine (Ozias-Akinns et al., 1993; Alvarez and Ordás, 2013). In orchid, when selection was delayed to 30 days no transformants were recovered, and the highest transformation efficiency was obtained when selection was initiated 2 days after transformation (Men et al., 2003b). The transformed EC were exposed to medium containing 400 mg l^-1^ 2-DOG as a selection agent. The 400 mg l^-1^ concentration of 2-DOG was used as it was reported to be the optimum concentration to kill untransformed oil palm EC (Masli et al., 2012). After exposure to selection medium containing 2-DOG, the untransformed EC began to die and allowing only the resistant calli to survive and form whitish embryoids within 6–10 months (**Figure 3C**). Compared to control, the calli started to grow well and proliferated into whitish and greenish embryoids (**Figure 3D**). The resistant calli were separated from dead untransformed calli to allow for better proliferation (**Figure 3E**). This is important because secretion of phenolic compound from dead adjacent cells could interfere and affect the regeneration process of transformed EC as demonstrated in strawberry (López Arnaldos et al., 2001).

After a few months, the whitish embryoids started to proliferate into greenish polyembryoid (**Figure 3F**) and subsequently developed into shoot buds on the selection medium (**Figures 3G,H**). The shoot buds were transferred into flasks to induce better growth of shoots (**Figure 3I**). After the desired shoots were obtained (∼5 cm long), the individual plantlets were transferred into test tubes containing RIM medium (**Figure 3J**). Plantlets with healthy shoots and roots were obtained within 4–6 months, and transferred into jiffy peat pellet to harden the plantlets (**Figure 3K**). Later, the plantlets were transferred into soil in polybags and kept in the screenhouse (**Figure 3L**). These plantlets were maintained according to standard nursery practices for oil palm. Taken together, it took nearly 2 years to regenerate transformed oil palm plantlets that were of approximately 15 cm in height from the day of transformation. Previously, Parveez et al. (2000) reported that it took almost 3 years to generate Basta-resistant transgenic oil palm plantlets after transformation using biolistic. The plantlets obtained were all phenotypically normal. This is not surprising as it has been reported previously that when potato was transformed with *DOG^R^1* gene, fertile and normal plantlets were obtained. This is because the exposure to 2-DOG is not expected to cause any alteration in plant metabolism due to the narrow substrate specificity of the enzyme (Kunze et al., 2001).

Based on the number of transformation events and final regeneration of transgenic oil palm, a transformation efficiency of 1.0% was obtained in this study when 2-DOG was used as a selection agent. Previously, when EC were selected on herbicide Basta, transformation efficiencies of 0.7–1.5% were reported (Parveez et al., 2000; Masli et al., 2009). However, the different in transformation efficiency has not been statistically compared. Generally, in this study, the used of 2-DOG could not further improve the transformation efficiency of oil palm. It was reported that different concentrations of kanamycin, hygromycin, phosphinotricin, and glyphosate were found to be effective to be used as selection agent for chickpea (Öz et al., 2009). However, transformation efficiency was suggested could be increased by introducing mechanical injury prior to transformation and followed by vacuum during bacterial inoculation. It was reported for soybean transformation, replacing kanamycin based selection to herbicide bialaphos based selection [also used the Basta resistant gene (*bar*)], managed to overcome the problems of chimeric shoots formation and high genotype dependency (Zhang et al., 1999). However, it still failed to improve the transformation efficiency. Later it was reported that adding various thiol compounds in the co-cultivation medium significantly increased the transformation efficiency of soybean (Olhoft and Somers, 2001; Olhoft et al., 2001; Zeng et al., 2004). In crambe, (*Crambe abyssinica*), it was reported that the transformation efficiency was similar when transformants were exposed to either constant low selection pressure (continuously on 3 mg/l hygromycin) or exposed to higher concentration and followed by lower selection concentration (started by 10 mg/l and followed by 3 mg/l of hygromycin; Li et al., 2013). This observation is in contradiction to many reports where higher transformation efficiency is obtained by gradually increasing the selection agent from low to higher concentration. Potrykus (1991) has suggested that one of the bottlenecks of plant transformation is in obtaining high transformation efficiency or higher rate of obtaining stable transformation in comparison to transient expression. Furthermore, calculation of transformation efficiency is very subjective as it is dependent on the type of explant. Rosellini (2012) considers transformation efficiency as the number of independent transgenic events obtained from 100 explants. This is quite easy for explants such as immature embryos or embryogenic axes (Aragão et al., 1996). However, for oil palm, as EC were used, the calculation of transformation efficiency was based on percentage of stable transformation events over the number of transiently expressing cells per plate (a mean of 100 transient expression per plate; Parveez et al., 2000).

### Molecular Characterization of 2-DOG Resistance Transgenic Oil Palm

**FIGURE 4:**
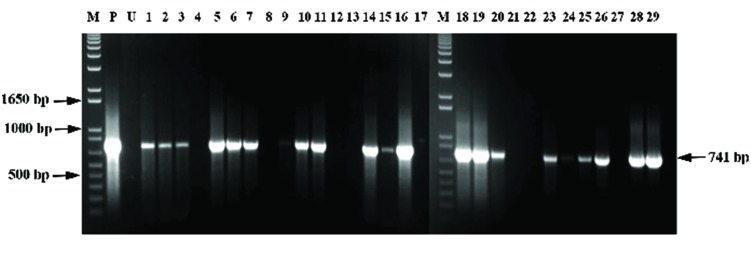
**PCR analysis for detection of *DOG^R^1* gene, PCR product is 741 bp**. Lane M, 1 kb plus marker; Lane P, pBIDOG plasmid; Lane U, untransformed sample; Lanes 1–29, transformed oil palm embryoid samples.

Regeneration of transgenic plants that are able to survive on 2-DOG provides initial evidence that the plants are transgenic; however, molecular analyses (PCR and Southern hybridization) are required to confirm stable integration of transgenes in plant genome. In this study, DNA from resistant embryoids was subjected to PCR analysis for the *DOG^R^1* gene. DNA from untransformed embryoids was also used as negative controls. Prior to performing PCR using the *DOG^R^1* gene, amplification of an internal control, specific to oil palm, was carried out (data not shown). A pair of primers (POR12 and POR38) which would specifically amplify a ∼1.1 kb size fragment of oil palm genomic DNA (Nurfahisza et al., 2014) was used as internal control. It is important that all samples (transgenic and negative control) used in this study amplified the 1.1 kb fragment before being used to amplify the transgene. A total of 29 putative transgenic embryoid samples and one untransformed embryoid sample were subjected to PCR analysis for the *DOG^R^1* gene. It was observed that 21 out of the 29 samples tested were positive for *DOG^R^1* gene as the expected band size of 741 bp was successfully amplified (**Figure 4**). No amplification of the band was observed for the untransformed negative control. Based on the results, it could be estimated that around 72% of the samples carried the *DOG^R^1* gene in their genome.

**FIGURE 5:**
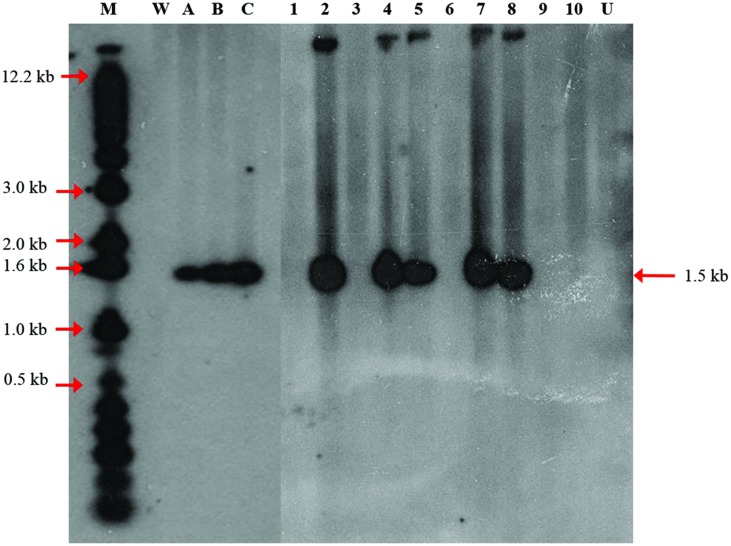
**Integration patterns of the *DOG^R^1* gene determined by Southern blot analysis**. Thirty μg oil palm genomic DNA was digested with *Hind*III and *Eco*RI and blotted onto nylon membrane and probed with a dATP^32^ labeled 1.5 kb *CaMV35S-DOG^R^1-nos* fragment isolated from pBIDOG plasmid. Copy number reconstruction experiment was performed by loading 30 μg untransformed genomic DNA spiked with 0 (lane W), 0.1 (lane A), 0.5 (lane B), and 1 (lane C) copy of the 1.5 kb *CaMV35S-DOG^R^1-nos* fragment. Lane M, 1 kb plus marker; Lane U, untransformed plant; Lanes 1–10, samples of transformed oil palm shoots.

Polymerase chain reaction analysis of the transgene only provides initial evidence of the presence of the transgenes in the genome of putative transgenic oil palm. For definitive evidence, Southern blot analysis is required to demonstrate integration of transgene into the genome of oil palm. Genomic DNA from transformed plantlet leaves were digested with *Eco*RI and *Hind*III and subjected to Southern hybridization using *CaMV35S-DOG^R^1-nos* fragment (**Figure 1**) as probe. After hybridization, it was observed that only 5 out of 10 transformed DNA samples (randomly chosen) showed hybridization to the expected 1.5 kb band (**Figure 5**). No signal was observed on untransformed sample (Lane U). Based on the DNA digestion profile, the ∼1.5 kb band was expected to correspond to the *CaMV35S-DOG^R^1-nos* fragment. This hybridization with expected band size demonstrates the integration of the transgene. Based on the intensity of hybridizing band compared to the copy number reconstruction lanes (**Figure 5**, lanes A–C), it was estimated that only two transformed DNA samples (lanes 5 and 8) carried a single copy of the *DOG^R^1* gene while the other three DNA samples (lanes 2, 4, and 7) contained multiple copies of the *DOG^R^1* gene.

In this study, it was demonstrated that around 72% of the samples analyzed by PCR showed the integration of transgene. It was reported that when oil palm plantlets derived from transformation using Basta resistant gene (*bar*), around 90% of the plantlets regenerated showed the presence of *bar* gene after PCR analysis (Parveez et al., 2008, 2015b). It was also reported that for transformed oil palm selected on mannose, about 90% of embryoids and 63–66% of plantlets showed positive PCR for the *pmi* gene (Bahariah et al., 2013). When oil palm immature embryos were selected on hygromycin, only 4–13% of the plantlets tested showed PCR positive for the *hptII* gene (Bhore and Shah, 2012). It was also shown in other plants such as garlic, *Artemisia annua* and sweet potato that around 86–92% of the transgenic plants regenerated were tested positive for the transgene used for selection (Zang et al., 2009; Lagunes-Fortiz et al., 2013; Tian et al., 2013). Therefore, the possibility of escape plants regenerated after selection is common and PCR analysis could be a good early detection approach to remove the unwanted escapes. In this study, the presence of around 28% escapes was surprising as the selection of transformants using 2-DOG should be easy to differentiate between the transformed and untransformed calli as 2-DOG will kill the untransformed calli and make them blackish while the transformants remain fresh and whitish. Meanwhile, Southern blot result confirmed the integration and copy number of transgene in transgenic oil palm plants obtained. Two out of five transgenic oil palms contained single copy of transgene. This result is expected since the transgenic oil palms were produced using *Agrobacterium* mediated transformation which is generally known to have the tendency to produce transgenic plants with lower copy number of the T-DNA (Kohli et al., 2003).

## Conclusion

We described here an effort to increase the transformation efficiency of oil palm using a new selection system, 2-DOG as a selection agent. Transgenic plants were obtained and confirmed using PCR and Southern analyses. A transformation efficiency of around 1.0% was obtained, which is about similar to the previously reported transformation efficiency of oil palm using herbicide Basta as selection agent. As the efforts to date, using four different selection agents, failed to increase the transformation efficiency, it is thus proposed that optimization of some of the steps in transformation protocol (**Figure 2**), could be exploited to increase the transformation efficiency of oil palm. Strengthening the selection scheme might also help to reduce the escape plants.

## Author Contributions

AMDI established the protocol and drafted the manuscript. MYAM helped to construct the transformation vector and edited the manuscript. II provided advice and guidance throughout the study. GKAP initiated and guided the project, and final critically write up of the manuscript.

## Conflict of Interest Statement

The authors declare that the research was conducted in the absence of any commercial or financial relationships that could be construed as a potential conflict of interest.
